# GBERT: A hybrid deep learning model based on GPT-BERT for fake news detection

**DOI:** 10.1016/j.heliyon.2024.e35865

**Published:** 2024-08-06

**Authors:** Pummy Dhiman, Amandeep Kaur, Deepali Gupta, Sapna Juneja, Ali Nauman, Ghulam Muhammad

**Affiliations:** a Chitkara University Institute of Engineering and Technology, Chitkara University, Rajpura, 140601, Punjab, India; b Department of CSE (AI), KIET Group of Institutions, Ghaziabad, 201206, India; c School of Computer Science and Engineering , Yeungnam University , Republic of Korea; d Department of Computer Engineering, College of Computer and Information Sciences, King Saud University, Riyadh, Saudi Arabia

**Keywords:** Deep learning, Fake news detection, Internet access, Large language model, Social media, Technology, Text classification, Transformers

## Abstract

The digital era has expanded social exposure with easy internet access for mobile users, allowing for global communication. Now, people can get to know what is going on around the globe with just a click; however, this has also resulted in the issue of fake news. Fake news is content that pretends to be true but is actually false and is disseminated to defraud. Fake news poses a threat to harmony, politics, the economy, and public opinion. As a result, bogus news detection has become an emerging research domain to identify a given piece of text as genuine or fraudulent. In this paper, a new framework called Generative Bidirectional Encoder Representations from Transformers (GBERT) is proposed that leverages a combination of Generative pre-trained transformer (GPT) and Bidirectional Encoder Representations from Transformers (BERT) and addresses the fake news classification problem. This framework combines the best features of both cutting-edge techniques—BERT's deep contextual understanding and the generative capabilities of GPT—to create a comprehensive representation of a given text. Both GPT and BERT are fine-tuned on two real-world benchmark corpora and have attained 95.30 % accuracy, 95.13 % precision, 97.35 % sensitivity, and a 96.23 % F1 score. The statistical test results indicate the effectiveness of the fine-tuned framework for fake news detection and suggest that it can be a promising approach for eradicating this global issue of fake news in the digital landscape.

## 1 Introduction

In the digital age, information is easily accessible, but the credibility of information cannot be guaranteed. The internet openness allows for the unrestricted dissemination of information, leading to the spread of misinformation [1]. News, which refers to public awareness of events, has seen a significant increase in fake news. Individuals and official groups create and share phony information on social media and other platforms, which can be dangerous, mislead people, harm democracy, and cause violence [2]. This threat is so severe that the Macquarie Dictionary named "fake news" the word of the year in 2016, recognizing its insidious hold on collective awareness [3].

Fake news can be interpreted as "fake information disorder” the term introduced by The European Council research report [4]. It can manifest in various forms, such as misinformation, disinformation, rumor, and malformation. In developing countries like India, the topic is still emerging due to the widespread availability of mobile internet and lack of media literacy among those not well aware of internet usage. Social media services [5] like Facebook and Instagram have become de facto information hubs, making them easy targets for spreading misinformation. Death hoaxes are also widespread in the digital age, with former Zimbabwean cricketer Heath Streak denying death rumors and seeking an apology from the author for the incorrect material, highlighting the necessity of social media verification and accountability [6]. Technological advances have made digital media manipulation possible in ways no one could have anticipated 20 years ago [7]. This is the reason, Geoffrey Hinton, the "Godfather of AI," worries about misinformation from generative AI, stating that the internet will be full of fake photographs, videos, and texts, making it difficult for people to tell the truth.

In this technological era, content is generated more frequently, making manual detection tedious. Researchers and academics worldwide have developed automatic false news-detecting methods to solve this problem [8]. Although natural language processing (NLP) has made steady progress in the last several years [9], identifying false news remains an arduous task to analyze unstructured content and apply machine learning or deep learning models [10]. For effective text classification, the deep learning (DL) approach recurrent neural network (RNN) captures contextual information from sequential data but has limitations in capturing long-range dependencies. Transformers, another revolutionary DL architecture, introduced an attention mechanism that revolutionized model design and overcame RNN's limitations due to their amazing concept of self-attention.

Large Language Models (LLMs) [11], like Bidirectional Encoder Representations from Transformers (BERT) [12] and Generative pre-trained transformer (GPT) [13] use transformer architecture to master natural language comprehension and production, improving text classification. These models are the latest NLP approaches for many problems. However, these fake news-detecting methods are currently being studied. To the best of the current knowledge, limited work has been done on the integration of these cutting-edge techniques in identifying fake news. The primary objective of this study is to examine the effectiveness of LLMs in identifying fake news to address the underlying research question (RQ).

**RQ:** Does the transformer architecture fusion contribute to the accuracy of identifying fake news?

### 1.1 Method

The main purpose of the current work is to propose a model that leverages BERT's bidirectional context understanding and GPT's generative capabilities to achieve robustness and adaptability in identifying fake news. To tackle the challenge of identifying fake news, a hybrid model named GBERT (Generative Bidirectional Encoder Representations from Transformers) is introduced by fusing GPT and BERT. These two cutting-edge methods extract the semantic meaning of words in a given sentence, as in our case with the given news content. This richer data representation resulted in a classification accuracy of 95.30 % in comparison to other approaches explored in this study.

Here are some key contributions from this study.

• A novel framework, GBERT, for combining two cutting-edge language models, GPT and BERT, is proposed. The BERT model is employed to capture contextualized word embeddings from every token, while GPT captures global dependencies and semantic coherence in news articles. Both models combine to create a fused representation that encompasses a wide range of linguistic features essential for distinguishing between authentic and fraudulent news.
• The proposed framework consists of three steps. After utilizing the best features of BERT and GPT, in the second step, the integration of BERT and GPT output takes place. The final step is to input this fusion into a dense network, which finally produces the classification of news as real or fake.
• The proposed GBERT framework is evaluated on two real-world corpora and compared to various ML and DL techniques.

### 1.2 Outline

This study is organized into the following parts: The "Related Work" section (Section 2) presents a review of existing AI techniques used to identify false news. Section 3 describes the problem formulation, architecture, and algorithm of the proposed framework. Section 4 outlines the experiment setup, pre-processing, evaluation criteria, and fine-tuning used to conduct the proposed approach. Section 5 presents the acquired findings of the experiment. This part also does a comparative analysis. Finally, Section 6 brings this research to a close by stating limitations and future recommendations.

## 2 Related work

In this section, the related work carried out by researchers is emphasized. Researchers and academicians have come up with many ways to spot fake news in order with the objective of mitigating its dissemination and preserving the credibility of the digital landscape. The exact meaning of fake news can be complex, as different researchers have different ideas about it as displayed in Table 1.

**Table 1:** Various definitions of "fake news" are used by various researchers.

Reference	Fake News Definition
W. Ansar and S. Goswami [14]	“Projected as an umbrella-term which encompasses all other associated terms such as misinformation, disinformation, fabricated news, satire, rumor, hoax and so on.”
K. Shu et al. [1]	“It is a news article that is intentionally and verifiably false.”
N. Belloir et al. [15]	“It is false but verifiable news composed of false facts based on real ones. Drafted in a way to trigger an emotional load, it aims to deceive its readers and in- fluence their opinion through an implicit conclusion.”
The Merriam Webster Online Dictionary [3]	“News reports that are intentionally false or misleading.”
O. Ajao et al. [16]	“Any story circulated, shared or propagated which cannot be authenticated.”
A. Dhawan et al. [17]	“Fabricated information created with an intent to cause damage to an individual or organization or to mislead people.”
P. Dhiman et al. [4]	“It pertains to the intentional or unintentional dissemination of counterfeit information meant to deceive or mislead, as well as create confusion, tension, and disbelief.”

**Fig. 1:**
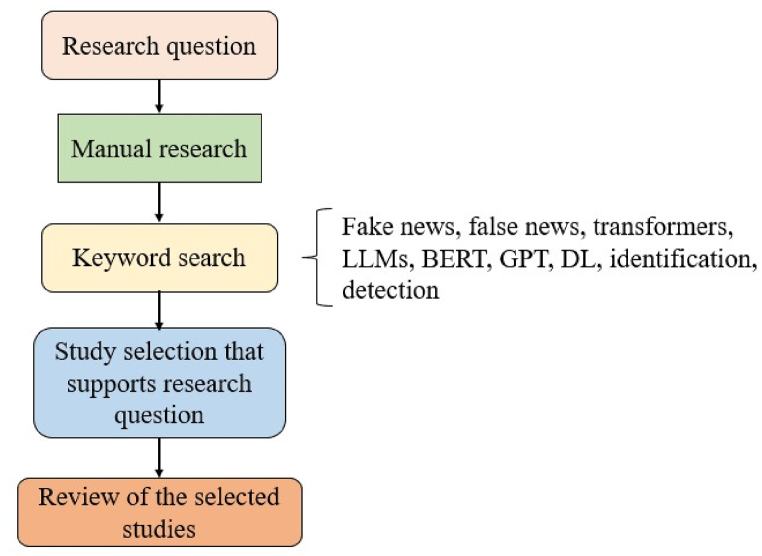
Article selection process.

**Fig. 2:**
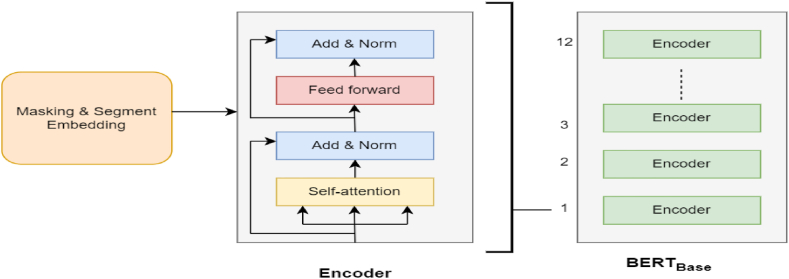
Bert processing layer.

**Threats to validity:** To delve into the research work conducted by various researchers, studies that deal with machine learning (ML), DL, and transformers for fake news detection (FND) are included in this work [18,19]. This study involves traditional search, employing specific keywords such as ‘fake’, ‘news’, ‘detection’, ‘identification’, ‘classification’, ‘transformers’, ‘BERT’, ‘GPT’, and ‘DL’. Literature research is conducted using various database resources, including Scopus, IEEE Xplore, ScienceDirect, the ACM Digital Library, and SpringerLink. Subsequently, papers with pertinent titles and abstracts are selected, and an evaluation takes place. Fig. 1 depicts the literature selection process employed in this study (see Fig. 2).

This section is broken down into research conducted based on the ML approach and exploring the impact of sentiment [20,21] in the propagation of false news. It also encompasses studies rooted in the DL approach [22]. Lastly, it delves into research conducted utilizing LLM.

### 2.1 Machine learning approaches in FND

This sub-section highlights the related work conducted by researchers using ML approaches in identifying fake news. In Ref. [23], authors detected fake news on social media posts using a Naïve Bayes classifier and achieved 74 % accuracy. According to Ref. [24], predictive analysis techniques also played a major role in fake news identification. Data cleaning through pre-processing, feature extraction, and then classification are the three main pillars of detecting counterfeit news content. The authors have developed a hybrid model by combining K-Nearest Neighbors (KNN) and Random Forest, and they have achieved an 8 % improvement in recognizing fake news.

### 2.2 Role of sentiment in fake news propagation

Several research papers have suggested utilizing sentiment analysis [25] as a means of identifying deceit, as there exists a potential association between the sentiment conveyed in a news piece and its veracity. Emotionally engaging content is more likely to become viral [26]. In Ref. [27], authors proposed a DL methodology to detect false news using news headline body pairings. The authors used BERT to extract uniqueness and emotion-aware representations [28], and a logistic regression model for categorization. Experimental findings demonstrated that the proposed model is more effective than previous methods at identifying bogus news. In another study [29], the authors used sentiment screening of news articles and emotion evaluation of user comments on those articles for feature extraction [30]. The Fakeddit dataset was utilized for this purpose. The proposed bidirectional long short-term memory (Bi-LSTM) was utilized to detect fake news by incorporating the content element of the news, in addition to the aforementioned features. This integration resulted in a notable detection accuracy of 96.77 %.

### 2.3 Deep learning approaches in FND

Deep learning techniques show potential for distinguishing between genuine and bogus content. The authors in Ref. [30] proposed an AI-based system incorporating NLP to recognize fake news. The results indicated that Random Forest and convolutional neural networks (CNN) with GlobalMaxpool performed exceptionally well. DL demonstrated a 6 % accuracy improvement in recognizing counterfeit news as compared to ML techniques. Authors in Ref. [31] introduced a benchmark dataset specifically designed to detect false news from an Indian perspective. In the domain of text-only identification, the Random Forest algorithm demonstrated a notable accuracy rate of 94 %. Conversely, inside the realm of deep learning models, the Bi-LSTM architecture earned a slightly lower accuracy of 92.7 %. In the task of image classification, the Resnet-50 model demonstrated a peak accuracy of 70.8 %. During the investigation of multi-modality using the fusion of LSTM and VGG16, the highest achieved accuracy was 66 %. The authors in Ref. [32] devised a method to examine the significance of the association between news text and visual content in the prediction of fake news. The utilization of a Text-CNN, augmented with a supplementary fully connected layer, is employed to generate textual representations. In contrast, for images, visual data is processed through the utilization of a pre-trained image2sentence model. The utilization of cosine similarity is employed to establish the degree of significance between textual and visual news. The F1 scores obtained from the PolitiFact and GossipCop datasets were 89.6 % and 89.5 %, respectively. Fake news also affects a country's economy because it has a direct impact on the stock market. To identify financial fake news, the authors in Ref. [33] collected 8k news samples related to the stock market. The experimental model based on CNN-LSTM achieved 92.1 % accurate results.

### 2.4 Language models in FND

A large language model (LLM) is an AI model that is designed to comprehend and generate human language. Various researchers opt to utilize these methodologies for categorizing fabricated information. The authors in Ref. [30] have conducted experiments to explore the usage of the transformer technique in addressing the issue of bogus news. The researchers put out a triple-branch BERT network as a solution for conducting binary and multi-label classification of fabricated news. The deployment of two corpora, namely LIAR and LIAR PLUS, resulted in a notable enhancement in accuracy for binary classifiers when compared to previously examined models. However, the improvement in accuracy for multi-label classifiers was only minimal. Authors in Ref. [34] employed an early fusion methodology to categorize fabricated news articles inside the Fakeddit dataset. The multimodal technique, which is based on CNN, integrates both textual and visual input with a precision rate of 87 %. Regarding unimodal methodologies, BERT exhibits a precision rate of 78 %. Authors in Ref. [35] presented a proposed hybrid model that utilizes BERT and LightGBM (light gradient boosting machine) to identify counterfeit news. The model aims to improve the accuracy of false news detection by leveraging the sophisticated linguistic pattern detection capabilities of BERT and the efficient feature space optimization and classification abilities of LightGBM. Authors integrated BERT and CNN to devise a novel message credibility (MCred) model to capture both global and local text semantics [36]. On the WELFake dataset, MCred outperforms traditional machine learning models, achieving 97.65 % accuracy and a 1.36 % improvement over the best-performing model. In addition, it outperforms BERT-RNN and BERT-LSTM models on the same dataset, with an accuracy of 99.01 %. The authors presented a novel approach for detecting out-of-context (OOC) media in detecting cheapfakes [11]. Introducing a feature extractor based on the GPT3.5 Large Language Model (LLM) improved the detection accuracy of the baseline algorithm, COSMOS. The GPT + AdaBoost classifier obtains the highest accuracy (89.4 %) and demonstrates superior generalization ability [37]. Table 2 provides a summary of the research conducted by various researchers around the globe.

**Table 2:** Literature review findings summary.

Year, Reference	Mechanism	Deals in	Dataset	Performance Analysis
2017 [38],	LSVM	Text	25200 news articles	92 % accuracy
2017 [39],	SVM	Text	BuzzFeed, Burfoot, and Baldwin	71 % accuracy
2018 [40],	Decision Tree	Text	613033 tweets	88 % F1 Score
2018 [41],	CNN, VGG19	Text, Image	Twitter, Weibo	82.7 % accuracy, 84.7 % precision, 81.2 % recall, and 82.9 % F1 score
2019 [16],	SVM, HAN	Text	PHEME	86 % accuracy
2020 [23],	Gaussian Naive Bayes	Text	538 Bangla news instances	87 % accuracy
2020 [32],	CNN, image2sentence	Text, Image	PolitiFact and GossipCop	89.5 % F1 score
2021 [31],	Random Forest, LSTM, Bi-LSTM,	Text,	IFND	Accuracy 94 % on RF, 92.7 % on Bi-LSTM
2021 [42],	BERT	Text	LIAR, LIAR PLUS	Binary classification (74 % accuracy, 69.9 % precision, 85.4 % recall, and 76.8 % F1 score)
2021 [33],	CNN-LSTM	Text	8000 samples	92.1 % accuracy
2022 [30],	Random Forest, CNN	Text	Kaggle	Accuracy 93 % on RF, 98 % on CNN
2022 [43],	BART	Text	NELA-GT-19 and Fakeddit	74.8 % accuracy, 72.4 % precision, 77.6 % recall, 74.9 % F1 score
2022 [10],	CNN, LSTM, BiLSTM	Text	9119 tweets	84.82 % accuracy
2022 [44],	DBN, RBM	Image	CASIA-WebFace, FFHQ, DFFD, 100K-Faces	97.82 % accuracy
2022 [45],	Naïve Bayes, LR, LSTM	Text	Hindi Fake and True Dataset	92.36 % accuracy
2022 [46],	Capsule neural network, BERT	Image, Text	PolitiFact and GossipCop	93 % accuracy on PolitiFact and 92 % accuracy on GossipCop
2023 [35],	BERT, LightGBM	Text	FNC	99.06 % accuracy,
2023 [37],	GPT-3	Text	ISOT	99.90 % accuracy, 99.81 % precision, 99.99 % recall, 99.90 % F1 score
2022 [36],	CNN, BERT	Text	Kaggle	99.01 % accuracy
2023 [11],	GPT-3.5, AdaBoost	Cheapfakes	ICME’23 Grand Challenge	89.4 % accuracy

Table 2 provides us with specifics.

• Researchers used ML and DL methods to capture the local context of a given text for fake news identification. However, the global context is not captured here; to address this, researchers turned to transformers to capture the global context.
• Model performance varies across approaches and datasets, with accuracy rates ranging from 71 % to 99.90 %.
• Advanced models, such as BERT, GPT, and hybrid architectures, have made significant progress in this field, as evidenced by their impressive performance metrics.
• Although LLMs have demonstrated considerable success in detecting bogus news, their utilization is still a topic of research.

In this paper, we propose a hybrid model by employing cutting-edge LLM models (BERT and GPT). These models excel at understanding contextual nuances and linguistic patterns in text, enabling more accurate detection of fake news. By leveraging BERT's bidirectional context understanding and GPT's generative capabilities, the proposed hybrid model contributes to identifying fake news.

## 3 Proposed GBERT model

Prior to looking into the architecture of the proposed framework, problem formulation is performed to provide a clear understanding of the objective of the methodology. In the context of fake news identification, the problem is characterized as a supervised task with the aim of determining whether a particular news item is fake or authentic.

### 3.1 Problem definition

Given a corpus of text news articles T and the task is to develop a binary fake news detection model. Mathematically the classification problem can be formulated as.

Let T = {A1, A2, …, An} represents n news articles comprising only of textual information and L € {0,1} represents news articles labels, where
0 implies that the news article is real
1 denotes that the news article is fake
The aim is to model a prediction function F that takes the feature vector 'X(A)' of a news article as an input and predicts the article's label, i.e., F(A) → {0, 1},
where:
F(A) = 0 if article 'A' is predicted as real
F(A) = 1 if article 'A' is predicted as fake

Recently, the transformer model has gained significant attention as a prominent advancement in DL within the field of NLP. This study proposes a hybrid deep learning framework, combining the strengths of BERT and GPT, to examine the effectiveness of transformers in identifying fake news. The architecture consists of three components, namely BERT, GPT, and the dense network layer. Here is a detailed breakdown of the architecture.

**Fig. 3:**
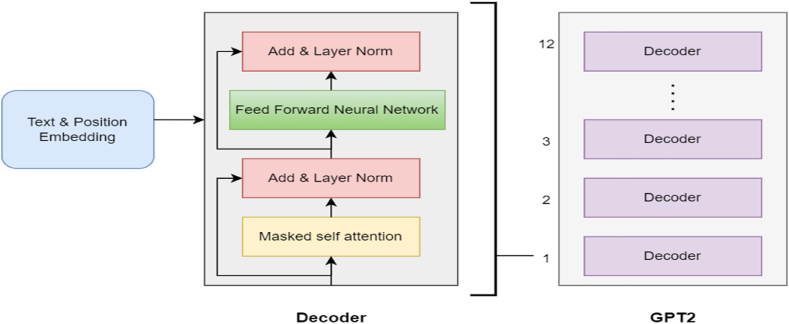
Gpt processing layer.

Bidirectional Encoder Representations from Transformers (BERT) Component:

BERT is a popular LLM that has undergone the extensive training on Google's enormous corpus to have a deeper understanding of language context. It is a machine learning framework that employs transformer neural network architecture [36]. If we breakdown the entire form of BERT, each term can be interpreted as follows (Fig. 3):

**Bi-directional:** As a context-dependent model, it can read text input in both left to right (LTR) and right to left (RTL)directions simultaneously using bidirectional methods [38].

**Encoder Representations:** As illustrated in Fig. 3, the encoder is made up of several layers of self-attention and feed-forward neural networks. It is crucial to the ability of the model to perceive semantics and their correlation in a given sentence.

**Transformer:** The Transformer architecture functions as the model's backbone and enables BERT to process and comprehend natural language text effectively [33]. It enables the model to extract contextual information from input text, efficiently manage variable-length sequences, and acquire robust word representations through self-attention. Attention is used to establish relationships between output and input components, facilitate the model to evaluate the significance of each word in a sentence relative to others [20].

There are two versions of the BERT model available, namely BERT_Base_ and BERT_Large_, differentiated by the number of layers used. This study made use of the BERT-base-uncased model, characterized by 12 layers and 768 hidden units per layer due to resource constraints. The pooler output, which is the second output, is selected. The variable B_output is assigned the responsibility of generating a summary of the contextual information included inside the input sequence.

### 3.2 Generative pre-trained transformer (GPT) component

GPT is a powerful language model that can identify contextual relationships and long-range dependencies in text, making it an efficient choice for various NLP tasks [37]. It learns to predict the subsequent word through unsupervised pre-training on large amounts of text data, acquiring a comprehensive understanding of grammar, semantics, and context. This unsupervised pre-training is the foundation for transfer learning, allowing GPT to be fine-tuned for specific tasks using smaller labelled datasets. GPT-2 is a Transformer architecture whose magnitude (1.5 billion parameters) was notable upon its release [21].

**Fig. 4:**
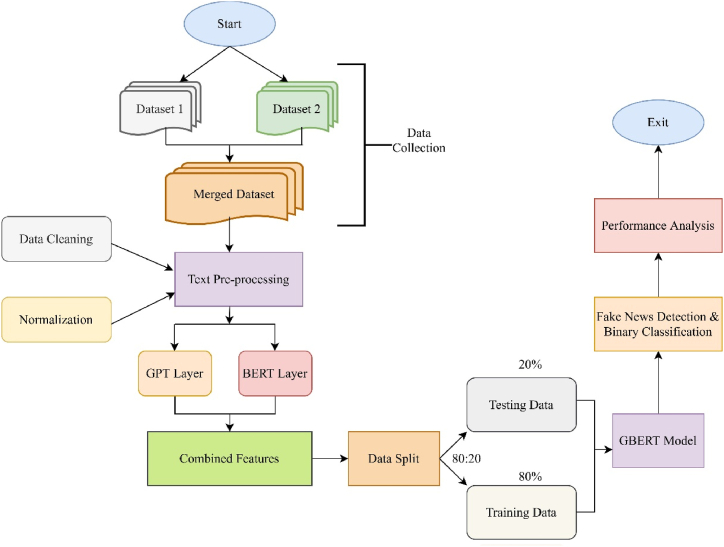
Block diagram of the proposed GBERT Framework.

GPT architecture is similar to the decoder part of transformer architecture (Fig. 4), outputs one token at a time, and then adds it to the sequence of input until the end of the statement is reached [37]. GPT uses masked self-attention, allowing it to peak at future tokens while processing each token to capture the relationship between them. GPT-2 architecture has several versions, each with different decoder counts and dimensionality (Table 3). In current work, GPT-2 model (small) is employed because of the limitations of the available resources. In order to achieve precision, the last hidden state of the last token is retrieved and afterwards set to the variable " G_output ".

**Table 3:** GPT-2 model specifications.

GPT 2	Small	Medium	Large	Extra Large
No of Decoder	12	24	36	48
Dimensionality	768	1024	1280	1600

The computational complexity of BERT and GPT is O(N*L^2^*H), where N represents the batch size, L shows the selected maximum sequence length, and H is the hidden size. Hardware acceleration and model size are other factors that can affect computational complexity.

### 3.3 Dense network layer

The amalgamation of the BERT and GPT models forms a hybrid representation. Using the bidirectional context of BERT and the generative capabilities of GPT, this integration leverages the best of both models. Fig. 4 represents the block diagram of the proposed hybrid framework for phony news recognition.

**Fig. 5:**
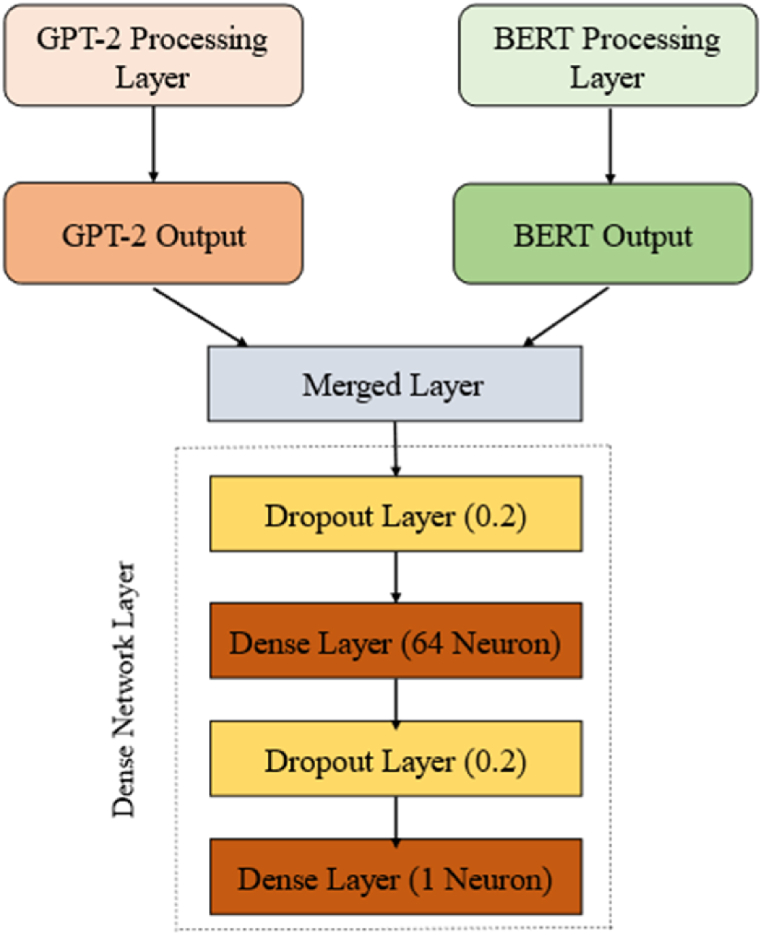
Dense network processing layer.

The procedure of merging the results obtained from the BERT and GPT2 models, denoted as Combine_input, is achieved through a concatenation layer. This fusion is subsequently passed to the dense network layer, which is a fully connected network of neuron layers (Fig. 5). Each neuron in a layer receives input from the neurons in the previous layer and transmits it to the neurons in the next layer. Each dense layer performs a dot multiplication of input and weight, while each hidden layer incorporates bias. These are then passed into the activation function, which determines how the weighted sum of the input is transformed into an output.

The Rectified Linear Unit (ReLU) activation function is implemented in inner layer. A supplementary dropout layer, with a dropout rate of 20 %, is incorporated after the dense layer, and sigmoid activation function is used in final layer, hence enhancing the model's ability to generalize. The optimization of the model is managed by the Adam optimizer, which is set up with a learning rate of 1e-05, epsilon of 1e-08, and gradient clipping norm of 1.0. The binary cross-entropy loss function plays a significant role in efficiently controlling the classification process with a high level of precision.

The algorithm outlines the steps involved in proposed method.

GBERT Model Algorithm.

**Table:** 

Data: Labelled Text Dataset
Result: Binary fake news detection model
Phase 1: Data Pre-processing
Dataset1 ←Collection (Text Dataset)
Dataset2 ←Collection (Text Dataset)
GBERT_dataset←Concatenate (Dataset1, Dataset2)
Phase 2: Model Generation
BERT
B_embeddings←BERT(GBERT_dataset)//Using bert-base-uncased pre-trained model
B_output←Pooled_output(B_embeddings)//Extract BERT's pooled output, which represents the entire input sequence
GPT
G_embeddings←G.decode(GBERT_dataset)//Decode text using GPT
G_ extract ←G_last_hidden_state(G_embeddings)//Capture the understanding of input text
G_output←G_extract(G_last_hidden_state)//Extract the final output representation from the last hidden state
Phase 3: Dense net processing layer
Combine_input←Merge (B_output, G_output)//Merge output from BERT and GPT processing layers
Final_output← Dense.dropout(Combine_input)//Combined input pass through dense and dropout layers
Binary_classification←label (Final_output)//Output is binary classified as fake or real

## 4 Experimental setup

The study is carried out with a 12th Generation Intel(R) Core (TM) i5-1235U processor functioning at a clock speed of 1.30 GHz. In order to carry out the experiment, the Kaggle platform is being leveraged. The experiment employed several essential Python modules, including sklearn.metrics for assessing classification metrics, matplotlib. pyplot for generating visualizations, and seaborn for improving the visual appeal of the plots.

### 4.1 Dataset

The experiment is carried out using two Indian benchmark data corpora. The first dataset is made up of news stories collected from different online news portals, while the news extracted from social media platforms comprise the second corpus. This section presents a comprehensive analysis of the two Indian corpora chosen for this investigation.

• **The Indian Fake News Dataset (IFND)** [31]**,** is a comprehensive dataset for detecting fake news in India, containing 37,809 real and 19,059 false news stories from 2013 to 2021. Although it doesn't include social media posts, it's crucial for studying misinformation and propaganda.
• **FakeNewsIndia Dataset** [17]**,** a benchmark corpus featuring false news with an Indian focus, includes 4803 instances of false news from 2016 to 2019 from six verified websites, providing insights into false news dissemination and countermeasures. Table 4 displays the statistical information derived from the combined dataset that was created by combining above mentioned datasets.

**Table 4:** Dataset statistics.

Dataset	Features		Label	Subjects	Statistics
IFND	News from mainstream news channels	Merged Dataset (IFND + FakeNewsIndia)	True News	Politics, Covid-19, Election, Violence and Miscellaneous	37800
FakeNewsIndia	News from YouTube and Twitter.		Fake News		23564

### 4.2 Pre-processing

Text data undergoes various steps of pre-processing to transform it into a relevant form for identifying fake news. Pre-processing techniques include folding all uppercase letters into lowercase, cleaning punctuation, special symbols, and URLs, removing stop words [47,48], stripping down text in square brackets and numbers, and removing emojis [49]. In NLP, en_US.utf-8 is used for accurate tokenization, proper sorting, and formatting of dates, times, and numbers according to American English conventions. Stemming and lemmatization techniques from NLTK are used for normalization within the dataframe and statement column. These techniques enhance text analysis by simplifying word variations, contributing to computational efficiency and linguistic accuracy [50].

### 4.3 Evaluation criteria

In this study, accuracy, precision, recall, and F1 score are used to compare the suggested model to existing approaches [51]. This research entails a binary classification analysis on bogus news, where the corpus is categorized into two distinct labels: fake and true.

Accuracy is the most common used performance measure.

(1) $$\text{Accuracy}=\frac{\left(TP+TN\right)}{\left(TP+FP+TN+FN\right)}$$

It is used to measure the effectiveness of the model in correctly identifying both fake news and true news out of the total number of news items as presented in Eq. (1) [52]. When there is imbalanced data, this performance metric can be misleading, potentially leading to a biased model that favors the majority labels [53]. As a result, performance metrics such as precision, recall, and F1-score become critical in order to provide a clearer picture of the model's realistic performance.

(2) $$\text{Precision}=\frac{TP}{\left(TP+FP\right)}$$

Eq. (2) represents the precision as the ratio of TP (predicted as false news and actually are fake news) to the sum of TP and FP (all news predicted as fake news). Recall value is also known as sensitivity, it evaluates the effectiveness of model to correctly recognize fake news instances as presented in Eq. (3) [54].

(3) $$\text{Recall}=\frac{TP}{\left(TP+FN\right)}$$

F1-Score is the harmonic mean of the two metrics precision and recall as depicted in Eq. (4). A high F1 score implies that the framework really identifies the target values (fake news) [55]. The F1-score ranges in value from 0 to 1. The highest value indicates strongest recall and precision balance.

(4) $$F1-\text{score}=2\times \frac{\left(Precision\times Recall\right)}{\left(Precision+Recall\right)}$$

### 4.4 Fine-tuning

Transfer learning is the masterstroke feature of LLMs that allows to use of a previously trained model to accomplish different jobs by fine-tuning them and enhancing their robustness against adversarial attacks [56]. In the current study, the complete architecture is used by using the pooler output of BERT, which summarizes the contextual information, and the last hidden state of GPT, which corresponds to the model's understanding of the given input text after processing the whole sequence. Then the architecture is fine-tuned by adding our dataset to the pre-existing datasets. Table 5 shows the details of the model fine-tuning parameters.

**Table 5:** GBERT model fine-tuning specifications.

Model	Parameter	Value
**BERT**	Number of dense layers	12
**GPT**	Number of dense layers	12
**Dense Network Layer**	Number of dense layers	2
	1st dense layer (neuron)	64
	Activation Function	ReLU
	2nd dense layer (neuron)	1
	Activation Function	Sigmoid
	Optimizer	Adam
	Loss Function	Binary Cross Entropy
	Batch size	16
	Number of epoches	3
	Dropout rate	0.2

## 5 Results and discussion

**Fig. 6:**
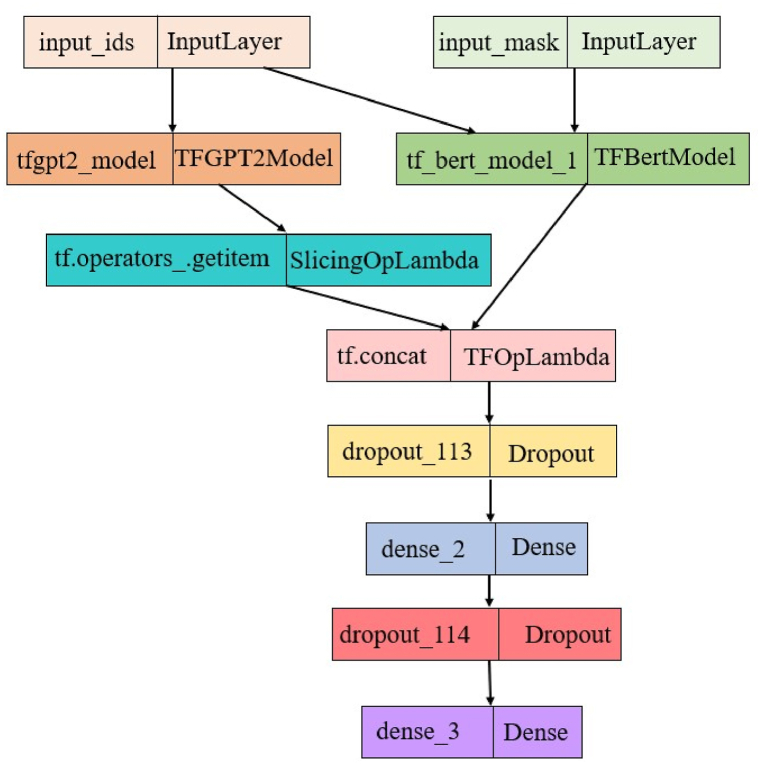
The architecture of proposed GBERT framework.

In this section, the results obtained by the GBERT model using the fine-tuning process presented in Sect. 4.4 are analysed. The present study combines the pooler output of BERT, which summarizes contextual information, with the last hidden state of GPT, which signifies the model's understanding of the input text after the entire sequence is processed. The GBERT architecture (Fig. 6) integrates BERT and GPT2 to create a syntactically balanced output. This research is conducted using an 80:20 train-test split.

To answer the research question, the proposed GBERT model, the first to integrate BERT and GPT, achieved an accuracy of 95.30 %, a precision of 95.13 %, a recall of 97.35 %, and an F1-score of 95.30 % in fake news identification.

**Fig. 7:**
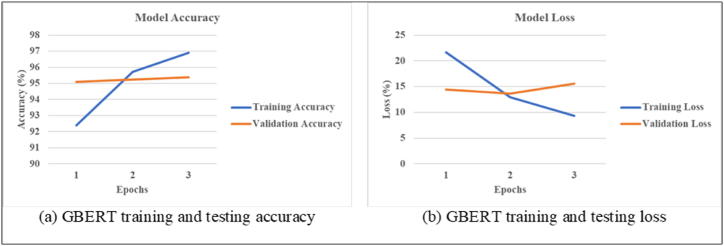
GBERT Learning Curve (a) accuracy (b) loss.

Fig. 7 (a) illustrates that with the rise in the number of epochs, training accuracy improves and testing accuracy stays relatively stable. The observed phenomenon, denoted as Fig. 7 (b), illustrates a gradual decline in training loss while an increase in testing loss as the number of epochs rises. This is because both models in this hybrid framework are complex. The model initially emphasizes learning from training data, but later it prioritizes better understanding the data, which results in better total accuracy despite some loss.

**Fig. 8:**
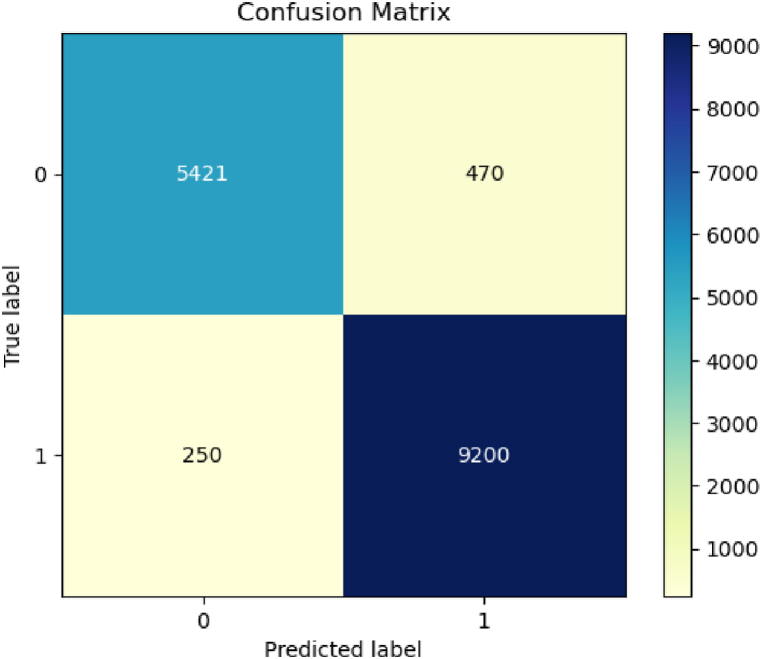
Confusion matrix for GBERT.

As shown in Fig. 8 high TP and TN values indicate that if the given statement is fake, it predicts fake, and if the input news is true, it predicts the same. In alternative terms, the model exhibits a high level of performance.

### 5.1 Comparative analysis of GBERT with ML models

Different ML methodologies like Multinomial Naive Bayes, pipeline comprising count vectorizer, TF-IDF, and Multinomial Naive Bayes and another pipeline comprising count vectorizer, TF-IDF, and Extreme Gradient Boosting (XGBoost), have been explored to determine their efficacy in the domain of false news identification. The TF-IDF method is used for feature extraction, which is then transformed into a feature vector. Table 6 displays the performance analysis of ML models employed in this work.

**Table 6:** GBERT performance comparison with other ML models.

Classifier	Accuracy (%)
MNB	92.03
MNB with pipeline	93.32
XGBoost with pipeline	93.42
Proposed (GBERT)	95.30

Table 6 shows that for the task of fake news detection, XGBoost outperformed other ML models by achieving 93.42 % accuracy. Yet it is lower than the proposed GBERT approach, demonstrating that the integration of two cutting-edge techniques improved the fake news recognition accuracy.

### 5.2 Comparative analysis of GBERT with DL approaches

To assess the efficacy of the presented strategy, the results are examined with other prevalent DL [57] and LLM methodologies utilized in the domain of fake news recognition. A pre-trained BERT model is loaded from the Hugging Face repository and trained using 80 % of the dataset. After tuning this LLM model, experimental results show BERT achieved 95.13 % accuracy, 95.21 % precision, 96.96 % recall or sensitivity, and a 96.08 % F1 score value. After that, this study also employed GPT-2 for this specific objective, and the obtained results are depicted in Table 7.

**Table 7:** GBERT performance comparison with other DL and LLM models.

Classifier	Accuracy (%)	Precision (%)	Recall (%)	F1-score(%)
BERT	95.13	95.21	96.96	96.08
GPT-2	95.00	94.11	94.11	94.08
Proposed (CNN + LSTM + LR)	94.19	95.05	95.54	95.29
Proposed GBERT	95.30	95.13	97.35	96.23

In some instances, the fusion of ML and DL also performs better. Thereby, a hybrid technique comprising convolutional neural networks (CNN), long-short-term memory (LSTM) networks [58], and logistic regression (LR) is presented to assess the GBERT model.

Table 7 shows that for the text classification in this current scenario of fake news identification, there is a marginal difference in precision between BERT and GBERT. This could be due to the architectural modifications or additional complexity. Despite this, GBERT outperforms all other implemented models in this work in other performance metrics for identifying subtle linguistic patterns and context-specific cues for splendid performance in bogus news recognition.

**Fig. 9:**
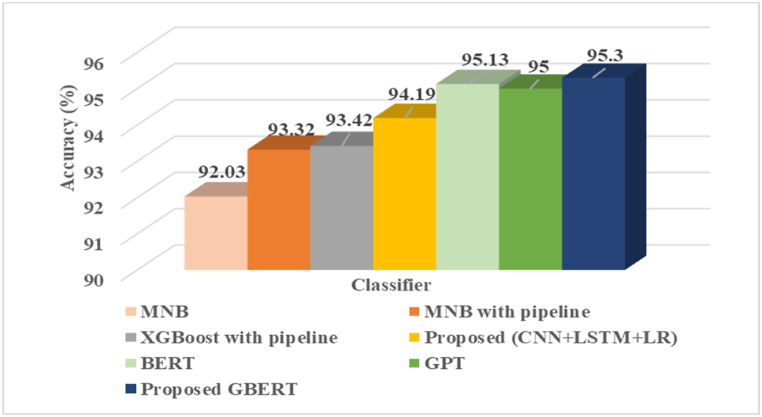
Graphical comparison of GBERT with different techniques.

Fig. 9 illustrates the visual comparison of the proposed framework with different techniques for better visualization and understanding.

### 5.3 Statistical significance

Table 7 presents the comparison of GBERT with various ML, DL, and LLM approaches. It is palpable that the proposed framework outperformed other models; however, there is a marginal difference between BERT and GBERT. To validate the significance of the proposed model's results [53], hypothesis testing is employed to determine whether there is significance in the results achieved between the model utilizing BERT only and the other combining BERT with GPT. For current work, hypothesis being tested is stated as.

• Null Hypothesis (H_0_): states that there is no statistically significant difference in performance between the BERT-only and GBERT model.
• Alternative Hypothesis (H_a_): There exists a statistically significant difference in performance between the BERT-only and GBERT model.

As there are two independent samples in this case (BERT-only model and GBERT model), an independent samples *t*-test is conducted. It measures how many standard deviations of the sample mean are away from the null hypothesis mean. The P-value represents the probability of observing a t-statistic as extreme as the one derived from selected data sample, under the assumption that the H_0_ is true. A P-value lower than the significant value (0.05) indicates the rejection of the H_0_. A lower p-value indicates stronger evidence against the null hypothesis, indicating a significant difference in performance between classifiers. If the p-value exceeds or equals the significant value, it signifies that the classifiers' performance does not significantly differ, thereby accepting the null hypothesis.

The achieved *t*-test value of −5.207 indicates a statistically significant difference in the mean accuracy between these two models, indicating that GBERT outperformed the BERT-only model. The achieved P-value of 0.0000001935, less than 0.05, indicates the rejection of H_0_. It means the improvement in performance is not random, but a meaningful enhancement.

Although statistical tests provide valuable insights to validate the significance of the proposed model's performance, it is essential to emphasize the factors that impose constraints [59].

• While the p-value indicates statistical significance, it's crucial to stress that the p-value alone cannot determine the correctness of a statistical test. It relies on various factors, specifically sample size, comparisons, data characteristics, and the format used to represent the final outcome.
• A larger sample size can result in an accidently significant p-value; conversely, a small sample size can result in a reduction in reliability. The presence of bias also impacts the findings, as the model uses the same biased data for training and testing purposes.
• Furthermore, contextual and model-specific factors such as architecture, hyperparameter tuning, and training data also have an impact on performance.

Therefore, it is crucial to thoroughly analyze and control these aspects to arrive at more significant conclusions about the models and their usefulness in real-life scenarios.

### 5.4 State-of-the-art comparison

Table 8 compares our proposed GBERT framework with the recent models that have been previously proposed in literature.

**Table 8:** Comparison of GBERT with state-of-the-art techniques. The values are in %.

Reference	Dataset	Technique	Accuracy	Precision	Recall	F1-score
G. Wu et al. [11]	ICME’23 Grand Challenge	GPT-3.5, AdaBoost	89.4	_	_	_
E. Essa et al. [35]	FNC	BERT, LightGBM	99.06	_	_	_
P. K. Verma et al. [36]	Kaggle	CNN, BERT	99.01	_	_	_
S. Raza and C. Ding [43]	NELA-GT-19 and Fakeddit	BART	74.8	72.4	77.6	74.9
B. Palani et al. [46]	PolitiFact and GossipCop	Capsule neural network, BERT	93(PolitiFact) 92(GossipCop)	_	_	_
D. Mehta et al. [42]	LIAR, LIAR PLUS	BERT	74	69.9	85.4	76.8
**Proposed GBERT**	IFND, FakeNewsIndia	BERT + GPT-2	95.30	95.13	97.35	96.23

Although the proposed model achieved good results in identifying fake news by leveraging global text semantics and the use of pre-trained BERT and GPT models, the GBERT model stands out from other approaches proposed by various researchers. However, there is still room for improvement. The following are the limitations of the presented hybrid system.

• The computational complexity [51] for BERT and GPT during training time can be expressed as O(L^2^ × d), where L is the sequence length and d represents the hidden size. Therefore, these models require more time while processing larger inputs.
• In this work, base versions of LLMs are employed due to resource constraints. For LLMs larger the parameters, the better the performance. Therefore, employing the enhanced version leads to improved performance.
• Input data also has a direct impact on the output of the model. Appropriately pre-processed data can yield more optimized results.

## 6 Conclusion and future recommendations

This research has proposed a novel hybrid framework for fake news identification. The proposed model combines two cutting-edge language models, BERT and GPT, to have a deeper and more nuanced contextual understanding of text. To assess the efficacy of the proposed model, the results are examined with other prevalent ML methodologies utilized in the domain of fake news recognition. The decision to integrate GPT and BERT is a strategic approach that capitalizes on the respective strengths of both models to accurately differentiate between genuine and counterfeit news articles. Furthermore, according to current knowledge, there is not much evidence that combines BERT and GPT to create a hybrid model for fake news detection. To evaluate the effectiveness of the proposed approach, the results are compared with several prevalent AI methodologies utilized in the field of fake news detection. The XGBoost pipeline achieved the highest accuracy at 93.42 %, slightly outperforming the Multinomial Naive Bayes (MNB) pipeline and the standalone MNB model. However, the GBERT model outperformed all ML models with an accuracy of 95.30 %. The BERT model demonstrated strong performance with an accuracy of 95.13 %, precision of 95.21 %, recall of 96.96 %, and F1-score of 96.08 %. GPT-2 achieved 95.00 % accuracy, 94.11 % precision, recall, and an F1-score of 94.08 %. The hybrid CNN + LSTM + LR model achieved 94.19 % accuracy, 95.05 % precision, 95.54 % recall, and a 95.29 % F1-score. GBERT outperformed these models with an accuracy of 95.30 %, precision of 95.13 %, recall of 97.35 %, and F1-score of 96.23 % highlighting its superior capability in identifying fake news by capturing subtle linguistic patterns and context-specific cues.

Although there are some limitations associated with the proposed approach that need to be addressed. Given that the statistical values such as a low P-value indicate a significant difference between model's performance, it is essential to consider the practical implications of these findings. An optimal P-value does not necessarily correspond to the real-world scenarios. Various factors including data diversity, model robustness, and computational efficiency play crucial roles in real-world applications. The high accuracy and other performance metrics achieved in controlled experimental conditions may not consistently indicate performance in diverse and dynamic real-world settings.

Future work aims to explore the advanced versions of LLMs to create a comprehensive system for fake news identification. While this work focuses on binary classification and unimodality, future work will also tackle multimodality and multiclass classification. Imbalanced data also posed an impediment for learning-based models, resulting in skewed performance. It is imperative to resolve this hindrance in order to improve reliability and effectiveness in real-world scenarios. Current work follows the black-box model concept; forthcoming efforts will incorporate explainable AI (XAI) to boost transparency. Further research is needed to validate the model's performance across different datasets and real-world scenarios to ensure its robustness and generalizability.

## Funding

The work was supported by the Researchers Supporting Project number (RSP2024R34), 10.13039/501100002383King Saud University, Riyadh, Saudi Arabia.

## Data availability statement

Data will be made available on request. Please request to P. Dhiman (email: pummy.dhiman@chitkara.edu.in).

## CRediT authorship contribution statement

**Pummy Dhiman:** Conceptualization. **Amandeep Kaur:** Investigation, Formal analysis, Conceptualization. **Deepali Gupta:** Writing – review & editing, Resources, Project administration. **Sapna Juneja:** Writing – review & editing, Writing – original draft, Supervision, Software. **Ali Nauman:** Visualization, Validation, Supervision, Software, Resources. **Ghulam Muhammad:** Writing – review & editing, Supervision, Funding acquisition.

## Declaration of competing interest

The authors declare that they have no known competing financial interests or personal relationships that could have appeared to influence the work reported in this paper.

## Abbreviations

Adam: Adaptive Moment Estimation
BERT: Bidirectional Encoder Representations from Transformers
CLM: Causal Language Modeling
CNN: Convolutional Neural Networks
GPT: Generative Pre-trained Transformer
KNN: K-Nearest Neighbors
LightGBM: Light Gradient Boosting Machine
LR: Logistic Regression
LLM: Large Language Models
LSTM: Long Short-Term Memory
NLP: Natural Language Processing
ReLU: Rectified Linear Unit
XGB: Extreme Gradient Boosting
